# Impact of family history of cancer on colorectal cancer screening: a propensity score-matched analysis from the Health Information National Trends Survey (HINTS)

**DOI:** 10.1186/s43046-023-00201-3

**Published:** 2023-12-11

**Authors:** Maxwell Akonde, Eric Mishio Bawa, Ottovon Bismark Dakurah, Rajat Das Gupta

**Affiliations:** 1 Department of Epidemiology and Biostatistics, Arnold School of Public Health, University of South Carolina, Columbia, SC USA; 2 Center for Bioinformatics and Computational Biology, Stellenbosch University, Stellenbosch, South Africa

**Keywords:** Colorectal cancer, Screening, Family history, Propensity score

## Abstract

**Background:**

Early detection of colon cancer leads to better survival outcomes. This can be achieved through colorectal cancer (CRC) screening. People with a family history of cancer (FHC) have increased risk of developing CRC. Increasing screening in this group will reduce CRC mortality. This study evaluated CRC screening in people with FHC.

**Methods:**

The study used data from the Health Information National Trends Survey (HINTS) 5, cycle 3. This is an annual cross-sectional survey with a nationally representative sample of American adults. The objective was to study the association between FHC and performing CRC screening. Propensity score matching was used to create a matched population with variables that constituted beliefs in cancer from the survey. Replication procedure, which is based on repeated sampling and allows for accurate computation of standard errors, was used for calculating statistical tests. Multivariable models were fitted in the matched population to assess the association between FHC and performing CRC screening.

**Results:**

People with FHC were 14% (*OR* = 1.14; 95% *CI*: 0.81–1.60) more likely to perform CRC screening than those without FHC, even though not statistically significant. Age in years (*OR* = 1.14; 95% *CI*: 1.12–5.27) had increased likelihood of performing CRC screening, while other races such as American Indians/Alaskan Natives (except African Americans) compared to Caucasians (*OR* = 0.49; 95% *CI*: 0.29–0.84) had significantly decreased likelihood of performing CRC screening.

**Conclusion:**

FHC was not significantly associated with having a colorectal cancer screening test. Public health advocacy should be directed towards increasing awareness of CRC screening among people with FHC.

## Introduction

Colorectal cancer (CRC) was the fourth most incident and the second most deadly cancer globally in 2018 [1]. CRC screening which has changed rapidly over the last three decades, from the use of stool for a fecal occult blood test to virtual tests such as the computed tomography colonoscopy, remains a better strategy at reducing the cancer burden. The United States Preventive Services Task Force recommends colorectal cancer screening for people between the ages 45–75 years, and the decision to be screened for people above 75 years is an individual one [2, 3]. Screening aids in the early detection of polyps with the potential to develop into cancer. Higher screening activities have been associated with a reduction in colorectal cancer mortality rates by about 52.4% from 2000 to 2015. While initial incidence was high, screening has resulted in about 25.5% reduction in colorectal cancer incidence [4, 5].

People with family history of CRC are at increased risk of developing colorectal cancer. This is influenced by hereditary and genetic factors such as familial adenomatous polyposis (FAP) and hereditary nonpolyposis colorectal cancer (HNPCC) [6–8]. Even though these factors confer a high lifetime risk of CRC, the number of cancers these account for is in the minority. In combination with environmental factors, a significant proportion of US adults with family history of CRC have an elevated risk of CRC [8].

Despite the importance of CRC screening, adherence remains lower than the Center for Disease Control and Prevention’s (CDC) goal of attaining 80% screening rates in adults aged 50 years and above in 2018 [8–10]. Additionally, the recommendations for people with family history of cancer (FHC) are not well established and vary from early screening and frequency of screening to targeting some racial and ethnic groups [4, 8, 11]. For example, in individuals who have a first-degree relative diagnosed with CRC before 60 years, the recommendation is for them to start colonoscopy screening before 40 years or 10 years younger than the age of the youngest person in their family who was diagnosed with CRC. For individuals with first-degree relatives who had hereditary nonpolyposis colorectal cancer (HNPCC), screening is recommended at 25 years and repeated every 1 to 2 years. In other hereditary conditions such as adenomatous polyposis syndromes, screening may start as early as 10 years [12]. There is the potential for further reducing CRC morbidity and mortality if screening in people with FHC is improved. However, there is a paucity of data or evidence on screening rates in people with FHC compared to the general population. This study evaluated the CRC screening rates in people with a FHC using advanced epidemiological methods to create a matched population by beliefs in cancer distributed between those with FHC and those without FHC. The study also examined the distribution of other sociodemographic factors between the two groups.

## Methods

**Fig. 1:**
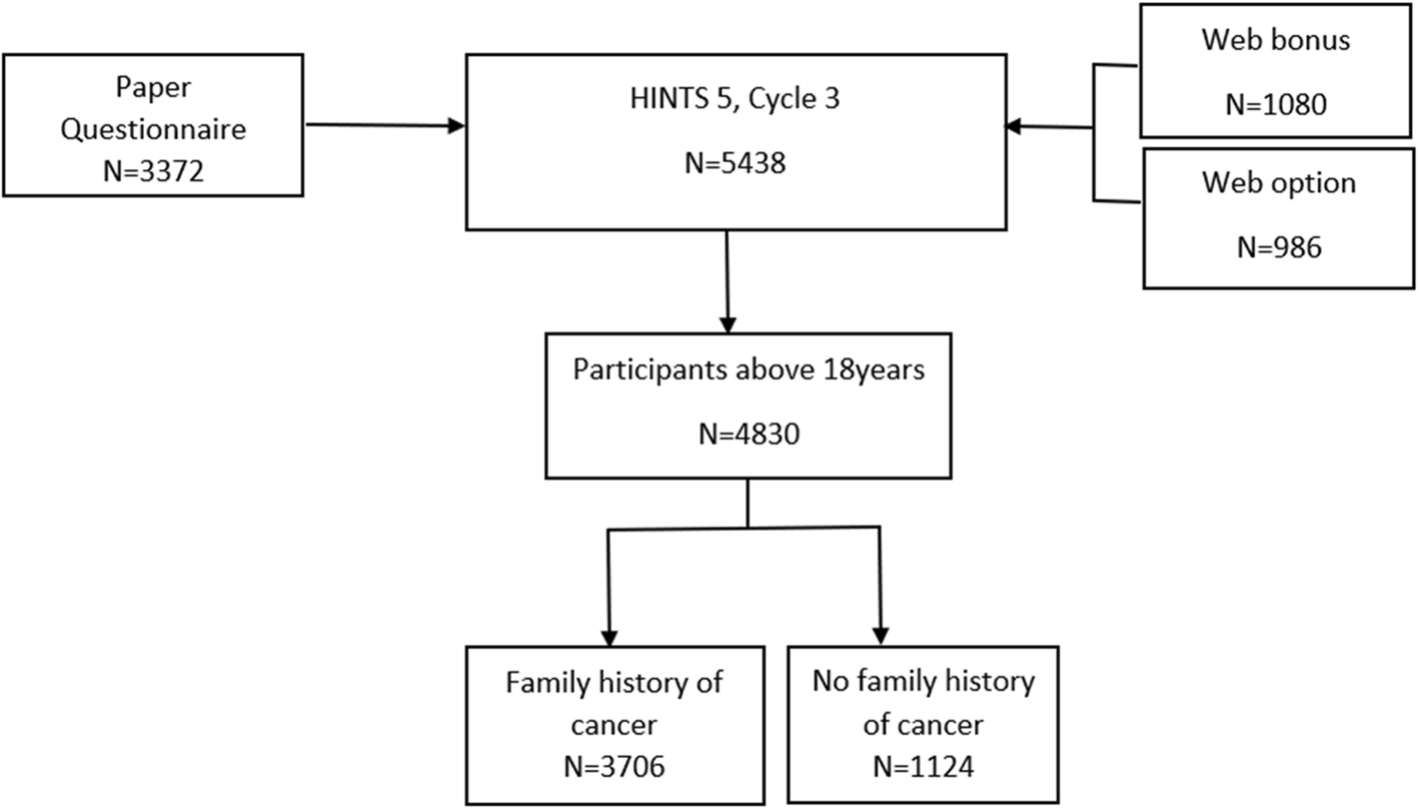
Summary of the data from the Health Information National Trends Survey (HINTS) 5, cycle 3 used in the data analyses

This study utilized data from the Health Information National Trends Survey (HINTS). HINTS is an annual cross-sectional survey of a nationally representative sample of American adults that assesses the impact of the health information environment. The study used the data from cycle 3 of HINTS 5. This data was collected between January 2019 and April 2019 and involved a self-administered mailed questionnaire and two experimental questionnaires conditioned in the Web Pilot. Participants in the Web Pilot were randomly assigned to either the Web Option, where participants had a choice of either responding via paper or the web, or web bonus, where participants chose either responding via paper or the web with a US $10 compensation. The detail of the survey design was previously published in the HINTS methodology report [13]. To provide a summary, all the conditions used the same sampling frame provided in the Marketing Systems Group (MSG) of addresses in the USA. The addresses were grouped into two strata comprising one stratum of addresses with high concentrations of minority populations and the other made of addresses with low concentrations of minority populations. All non-vacant addresses in the MSG database and seasonal addresses were subjected to the sampling. The addresses were divided into three representative subsamples to accommodate the web pilot: one for paper-only group, another for web option, and the third for the web bonus. The questionnaires were initially emailed, followed by a postcard reminder, and up to two additional mailings of the questionnaire as needed for non-responding households. The protocol for the web pilot was similar except the language of the cover letters differed based on whether the respondents were being invited to complete the paper or the web-based questionnaire. The second stage of the sampling procedure included selecting one adult within each sampled household using the next-birthday method. The questionnaire was made available either in English or Spanish, but for this study, we limited the data to only the English version. The HINTS 5, cycle 3 methodology report, provides more details on the sampling procedures and methods used [13]. A total of 5438 respondents formed part of HINTS 5, cycle 3, with 191 considered partial completers who did not answer the entire survey. A questionnaire was considered to be complete if at least 80% of sections A and B were answered. A questionnaire was considered to be partially complete if 50–79% of the questions were answered in sections A and B. The paper respondents were 3372, while 986 and 1080 responded with the web option group and web bonus group respectively. The cycle 3 overall response rate was 30.2% for the paper only, 29.6% for web option, and 31.5% for the web bonus, with the response rates not significantly different [14, 15]. We included all participants from the paper and web option who were 18 years and above. An unweighted total of 4830 people with 3706 who had a family history of cancer and 1124 without a family history of cancer were included as shown in Fig. 1. This is publicly available deidentified dataset and did not require further ethical approval. Additionally, all results were presented in aggregated formats to protect the data integrity and confidentiality of participants in accordance with HINTS data terms of use.

### Variables

#### Outcome

The main outcome variable was colorectal cancer screening test. This was assessed by asking the question “Have you ever had one of these tests to check for colon cancer?” with options “yes” or “no.” The outcome was dichotomized as yes and no: yes, for those who received a colon cancer screening test, and no, for those who did not receive a colon cancer screening test.

#### Main predictor

The main predictor was a family history of cancer. This was assessed by asking the question “Have any of your family members ever had cancer?” with the options “yes” or “no.” Family history of cancer was dichotomized as yes and no: yes, for those who had a family history of cancer, and no, for those who did not have a family history of cancer.

### Other variables

Other data that were measured included age (continuous), sex (male and female), sexual orientation (heterosexual, homosexual, and others), marital status (married, single, and divorced/separated), education (high school or less, some college, college graduate, and postgraduate), insurance (yes and no), smoker (never, current, and former), e-cigarette (ever and never), and race (African American, Caucasian, and others).

#### Beliefs in cancer

The following variables constituted beliefs of the participants in cancer: chances of getting cancer (How likely are you to get cancer in your lifetime?); everything causes cancer (How much do you agree or disagree: it seems like everything causes cancer?); not possible to prevent (How much do you agree or disagree: there is not much you can do to lower your chances of getting cancer?); too many recommendations on preventing cancer (How much do you agree or disagree: there are so many different recommendations about preventing cancer, it is hard to know which ones to follow?); unexplained bleeding as a sign of cancer (Do you think unexplained bleeding could be a sign of cancer?); change in bowel or bladder habits as sign of cancer (Do you think a change in bowel or bladder habits could be a sign of cancer?); obesity influences cancer (How much can obesity influence whether or not a person will develop cancer?); fiber influences cancer (How much can eating enough fiber influence whether or not a person will develop cancer?); processed meat influences cancer (How much can eating too much processed meat influence whether or not a person will develop cancer?); and fruit and vegetables influence cancer (How much can eating fruits and vegetables influence whether or not a person will develop cancer?).

Other variables considered as beliefs in cancer were trust information from doctor, getting a doctor’s text, seeking health information (Have you ever looked for information about health or medical topics from any source?), and ever had cancer (Have you ever been diagnosed as having cancer?).

### Data analysis

Data collected with paper only, Web Option, and web bonus were used for the analysis. We tested for group difference, and no significant group difference was found. Weighted analysis was performed. Weighted bivariate comparison of the demographic characteristics between those who had a family history of cancer and those without a family history was performed. Chi-square statistics were used to compare the categorical variables such as gender, and a *t*-test was used for the continuous variables such as age.

A propensity score matching was used to create a matched population that allows for both those with family history of cancer and those without family history to be comparable in terms of the variables that were used to create the scores. We created the propensity scores using the variables that constituted the beliefs about cancer in addition to trusting information from a doctor, getting a doctor’s text, seeking health information, and ever had cancer, thereby creating two comparable groups of people with family history of cancer and people without family history of cancer. The 1:1 propensity score-matched pair method combined with selected covariate adjustment for people with FHC and people without FHC was used. The PS matching yielded an unweighted 924 people with FHC and 924 people without FHC with no difference in the health beliefs in cancer, trust information from a doctor, getting a doctor’s text, seeking health information, and ever had cancer. We then fitted multivariate logistic regressions with colon cancer screening test as the outcome and family history of cancer as the predictor using the matched data. We also adjusted for variables that were not used in creating the propensity score matched data. We used the replication procedure, which is based on repeated sampling and allows for accurate computation of standard errors for the statistical testing procedures. The replicate weights were created using jackknife minus one replication method in the HINTS 5 cycle 3 with denominator degrees of freedom of 49. Details of the replication procedure are contained in the HINTS cycle 3 survey overview and data analysis recommendations document [14]. All analyses were done using the SAS/STAT® software version 9.4.

## Results

From Table 1 which presents the characteristics of the unmatched population, the average age of the population was 46.94 (0.87) years with those who had a family history of cancer (49.58 (0.29) years) being significantly older than those without a family history of cancer (48.19 (0.51) years) (*p*-value = 0.0162). Most of the participants had some college education (40.21%) and were females (51.28%). Gender (*p*-value = 0.0353), race (*p*-value = 0.0002), and cigarette use (*p*-value < 0.0001) differed significantly between those with a family history of cancer and those without a family history of cancer. There were 53.33% of participants without family history of cancer who were males compared to 47.21% of those with family history of cancer who were males. Most of the participants were Caucasians (77.8%), with 68.02% of those without family history of cancer being Caucasians compared to 80.93% of those with family history of cancer who were Caucasians. Almost two-thirds of the participants were never cigarette users. One-fourth of participants who had a family history of cancer were former cigarette users compared to 17.59% of participants who had no family history of cancer who were former cigarette users. In the unmatched population, the overall colon cancer screening prevalence was 47.19%. The prevalence of colorectal cancer testing among those with FHC was significantly higher (48.79%) compared to those without FHC (42.30%) (*p*-value = 0.0459).

**Table 1:** Characteristics of participants stratified by the family history of cancer in the unmatched sample

Variable	Unweighted				Weighted			
	**Total** ***N* (%)**	**No FHC** ***N* (%)**	**FHC** ***N* (%)**	***p*-value**	**Total** **%**	**No FHC** **%**	**FHC** **%**	***p*-value**
**Age, mean (SE)**	56.93 (16.89)	56.09 (17.11)	57.26 (16.69)	0.0407	46.94 (0.87)	48.19 (0.51)	49.58 (0.29)	0.0162
**Education**				0.5652				0.7439
< = High school	1114 (23.15)	265 (23.72)	849 (22.97)		29.30	29.28	29.30	
Some college	1437 (29.86)	322 (28.83)	1115 (30.17)		40.21	42.27	39.53	
College graduate	1297 (26.95)	315 (28.20)	982 (26.57)		17.69	16.24	18.16	
Postgraduate	965 (20.05)	215 (19.25)	750 (20.29)		12.81	12.21	13.00	
**Gender**				0.004				0.0353
Female	2803 (58.47)	600 (53.81)	2203 (59.88)		51.28	46.67	52.79	
Male	1991 (41.53)	515 (46.19)	1476 (40.12)		48.72	53.33	47.21	
**Marital status**								0.2934
Married	2634 (54.76)	620 (55.56)	2014 (54.52)	0.2672	56.38	56.92	56.20	
Separated	1387 (28.84)	302 (27.06)	1085 (29.37)		13.85	12.27	14.36	
Single	789 (16.40)	194 (17.38)	595 (16.11)		29.77	30.81	29.44	
**Race**				< .0001				0.0002
African Americans	704 (15.53)	188 (18.34)	516 (14.71)		12.51	15.61	11.52	
Caucasians	3409 (75.20)	681 (66.44)	2728 (77.77)		77.80	68.02	80.93	
Others	420 (9.27)	156 (15.22)	264 (7.53)		9.69	16.37	7.55	
**Sexual orientation**				0.2180				0.2019
Heterosexual	4359 (94.49)	1013 (95.30)	3346 (94.25)		93.76	95.51	93.19	
Homosexual	117 (2.54)	20 (1.88)	97 (2.73)		2.42	1.97	2.57	
Others	137 (2.97)	30 (2.82)	107 (3.01)		3.82	2.51	4.24	
**E-cigarette use**				0.0281				0.0525
Ever	640 (13.25)	128 (11.39)	512 (13.82)		19.31	14.98	20.73	
Never	4190 (86.75)	996 (88.61)	3194 (86.18)		80.70	85.02	79.27	
**Cigarette use**				< .0001				< 0.0001
Never	2971 (62.01)	756 (68.05)	2215 (60.19)		64.09	73.52	60.99	
Current	539 (11.25)	110 (9.90)	429 (11.66)		12.20	8.89	13.28	
Former	1281 (26.74)	245 (22.05)	1036 (28.15)		23.71	17.59	25.72	
**Colorectal cancer testing**				< 0.0001				0.0459
Yes	2949 (61.94)	620 (56.36)	2329 (63.62)		47.19	42.30	48.79	
No	1812 (38.06)	480 (43.64)	1332 (36.38)		52.81	57.70	51.21	

*SE* standard error, *N* number of participants, % percent of participants, *χ*^2^ chi-square statistic; age is in years, *FHC* family history of cancer

In the matched population in Table 2, the ages of those who had a family history of cancer (49.23 (0.58) years) and those without a family history of cancer (47.75 (0.56) years) were comparable (*p*-value = 0.0662). However, race significantly differed between the two groups. Among those without a family history of cancer, there were 13.37%, 70.56%, and 16.09% of African Americans, Caucasians, and other races compared to 15.49%, 77.92%, and 6.59% of African Americans, Caucasians, and other races in the participants with a family history of cancer group. There were slightly more males in the matched sample than females, even though this difference was not statistically significant across the different groups. In terms of cigarette use, 68.65%, 9.78%, and 21.57% of the matched participants were never, current, and former cigarette users respectively. In the matched population, even though the prevalence of colorectal cancer screening in those with FHC was higher (48.42%) compared to those without FHC (42.91%), this difference was comparable.

**Table 2:** Characteristics of participants stratified by the family history of cancer in the matched sample

Variable	Unweighted				Weighted			
	**Total** ***N* (%)**	**No FHC** ***N* (%)**	**FHC** ***N* (%)**	***p*-value**	**Total** **%**	**No FHC** **%**	**FHC** **%**	***p*-value**
**PS, mean (SD)**		0.29 (0.13)	0.29 (0.12)	0.6266		0.29 (0.13)	0.21 (0.11)	
**Age (years), mean(SE)**	56.49 (16.85)	55.83 (16.96)	57.15 (16.71)	0.0910	48.47 (0.40)	47.75 (0.56)	49.23 (0.58)	0.0662
**Education**				0.3297				0.5655
< = High school	410 (22.23)	195 (21.17)	215 (23.29)		27.70	25.34	30.22	
Some college	552 (29.93)	266 (28.88)	286 (30.99)		42.13	45.08	38.98	
College graduate	522 (28.32)	270 (29.32)	252 (27.30)		17.21	16.78	17.66	
Postgraduate	360 (19.52)	190 (20.63)	170 (18.42)		12.96	12.80	13.13	
**Gender**				0.0002				0.1968
Female	1054 (57.34)	488 (53.04)	566 (61.66)		49.44	46.66	52.44	
Male	784 (42.66)	432 (46.96)	352 (38.34)		50.56	53.34	47.56	
**Marital status**				0.0231				0.0733
Married	1026 (55.67)	534 (58.04)	492 (53.30)		57.19	58.90	55.35	
Separated	501 (27.18)	224 (24.35)	277 (30.01)		11.92	9.85	14.12	
Single	316 (17.15)	162 (17.61)	154 (16.69)		30.90	31.25	30.52	
**Race**				< 0.0001				0.0004
African Americans	285 (16.51)	136 (15.98)	149 (17.03)		14.39	13.37	15.49	
Caucasians	1234 (71.50)	583 (68.51)	651 (74.40)		74.14	70.56	77.92	
Others	207 (11.99)	132 (15.51)	75 (8.57)		11.46	16.09	6.59	
**Sexual orientation**								0.5705
Heterosexual	1675 (94.79)	837 (94.79)	838 (94.80)		93.57	94.74	92.33	
Homosexual	38 (2.15)	19 (2.15)	19 (2.15)		2.23	2.36	2.09	
Others	54 (3.06)	27 (3.06)	27 (3.05)		4.20	2.90	5.58	
**E-cigarette use**				0.6714				0.2659
Ever	228 (12.34)	111 (12.01)	117 (12.66)		18.86	16.56	21.32	
Never	1620 (87.66)	813 (87.99)	807 (87.34)		81.14	83.44	78.68	
**Cigarette use**				0.3722				0.0497
Never	1195 (65.19)	611 (66.63)	584 (63.76)		68.65	73.32	63.66	
Current	179 (9.77)	89 (9.71)	90 (9.83)		9.78	8.28	11.38	
Former	459 (25.04)	217 (23.66)	242 (26.42)		21.57	18.40	24.96	
**Colorectal cancer testing**				0.0013				0.1575
Yes	1095 (60.03)	512 (56.33)	583 (63.72)		48.37	42.91	48.42	
No	729 (39.97)	397 (43.67)	332 (36.28)		51.63	57.09	51.58	

*SD* standard deviation, *SE* standard error, *N* number of participants, % percent of participants, *χ*^2^ chi-square statistic; age is in years, *FHC* family history of cancer

In Table 3 which presents the results of the multivariable regression analysis, participants with a family history of cancer were 14% (*OR* = 1.14; 95% *CI*: 0.81–1.60) more likely to do a CRC screening test compared to those without a family history of cancer, even though not statistically significant. Again, even though participants with higher education were more likely to do CRC screening tests compared to those with high school education and below, education was not found to be a significant predictor. Single participants were 2.15 (*OR* = 2.15; 95% *CI*: 1.22–3.79) times as likely to do a CRC screening test compared to those who were married. Age was significantly associated with CRC screening (*OR* = 1.14; 95% *CI*: 1.12–5.27) as increasing age resulted in increasing odds of taking a CRC screening test. Participants with health insurance were 59% (*OR* = 1.59; 95% *CI*: 0.73–3.46) more likely to do a CRC screening test compared to those without health insurance, even though not statistically significant. Compared to Caucasians, other races were 51% (*OR* = 0.49; 95% *CI*: 0.29–0.84) significantly less likely to do a CRC screening test. Gender and sexual orientation were not significantly associated with CRC screening tests.

**Table 3:** Multivariable logistic regression of colorectal cancer testing using propensity score matching

Variable	Odds ratio (95% *CI*)
**Family history**	
No	1
Yes	1.14 (0.81–1.60)
**Education**	
High school	1
Some college	1.32 (0.75–2.33)
College graduate	1.37 (0.70–2.68)
Postgraduate	1.07 (0.56–2.07)
**Gender**	
Male	1
Female	1.16 (0.69–1.97)
**Race**	
Caucasian	1
African American	0.97 (0.47–2.04)
Other	0.49 (0.29–0.84)
**Cigarette use**	
Never	1
Current	0.56 (0.24–1.29)
Former	0.97 (0.49–1.89)
**E-cigarette use**	
Never	1
Ever	1.33 (0.66–2.70)
**Sexual orientation**	
Heterosexual	1
Homosexual	1.18 (0.23–5.99)
Other	0.56 (0.20–1.61)
**Marital status**	
Married	1
Separated	0.96 (0.57–1.64)
Single	2.15 (1.22–3.79)
**Age**	1.14 (1.12–5.27)
**Insurance**	
No	1
Yes	1.59 (0.73–3.46)

*CI* confidence interval

## Discussion

Family history of cancer is a known risk factor for CRC. While increased cancer screening is associated with improved cancer outcomes including reducing morbidity and mortality, cancer screening statistics in people with a family history of cancer are limited. This study examined colorectal cancer screening in people with a family history of cancer and how it compares to people without a family history of cancer. The prevalence of colon cancer testing among those with FHC was 48.79% compared to 42.30% in those without FHC with an overall screening rate of 47.19% recorded. Overall, CRC screening was 14% more in people with a family history of cancer compared to people without a family history of cancer even though this was not a significant difference. People who were single were 1.15 times more likely to perform a CRC screening compared to those who were married. This is likely a spurious association as divorce rates are increasing in the USA, and this population that was once married was identified either as single or separated [16]. Health insurance, education, gender, and sexual orientation were not significant predictors of CRC screening.

Previous studies found that CRC screening has been on the increase since the 2000s, and even though the USA missed the Healthy People 2020 (HP2020) target of 70.5%, screening rates were about 62.4–66.9% [17–19]. This rate was higher than the screening in the current study. This is most likely due to the differences in the age groups defined for the studies. While White et al. limited their study to people between the ages of 50 and 75 years [18], our study included people above the age of 18 years. This is likely to have created a larger population in our study with few people below the age of 50 years performing CRC screening. While CRC screening is recommended for adults over the age of 50 years, people with FHC are recommended, on the advice of their healthcare provider, to undergo CRC screening at ages younger than 50 years. For example, people with a first-degree relative diagnosed with CRC are recommended to undergo a CRC screening 10 years below the age at which the first-degree relative was diagnosed with the CRC, and for people with a first-degree relative diagnosed with HNPCC, CRC screening is recommended at 25 years. It is, therefore, important to examine CRC rates in people younger than 50 years when studying CRC screening in relation to FHC. The higher prevalence of CRC screening among people with FHC gives a positive outlook for the reduction in the burden of CRC in the US population. However, a value of 48.79% remains far below the HP2020 target [17, 19]. Additionally, after matching health beliefs in cancer and adjusting for race, age, education, health insurance, and gender among other variables, CRC screening did not vary between people with FHC and people without FHC. Beliefs in cancer are more likely to drive CRC screening [20, 21] than FHC. Focusing on creating awareness to impact more positive health beliefs and knowledge about cancer could induce attitudinal change and boost CRC screening rates.

Racial disparity in health outcomes has been an important topic. Our study found that other racial groups such as American Indians/Alaska Natives and Asian Indians (except African Americans who were 3% less likely to do a CRC screening test even though not significant) were 51% significantly less likely to receive a CRC screening test compared to Caucasians. White et al. and Sabatino et al. reported similar results. Sabatino et al. found that lower test receipt was associated with age 50–64 years, American Indian/Alaska Native or Asian race [19], while White et al. reported that colorectal cancer screening use was lowest among American Indians and Alaska Natives by racial group and lower screening test use in Hispanics compared to non-Hispanics by ethnicity [18]. Race and ethnicity have been a proxy measure for many health inequities including access to healthcare and related services. The lower test receipt observed in these races and ethnicities is likely due to lower access to CRC screening and lack of health insurance [22, 23]. Even though health insurance was not associated with receiving CRC screening, our study did not examine health insurance disparity across the different races and ethnicity in our study population.

The study has some notable limitations worth discussing, despite the important findings. First, the HINTS database is a cross-sectional study. The information collected was self-reported. This presents some bias situations such as reported bias and recall bias. These biases are likely nondifferential due to the robust and random data collection steps that were used. The use of the propensity score matching to create the matched data ensured the characteristics of those with a family history of cancer were compared with those without FHC and hence minimized residual confounding. Second, data on the specific types of CRC screening tests that were done were not reported. We were not therefore able to assess group differences between different CRC screening tests. Third, due to the cross-sectional nature of the data, it is difficult to establish any temporal sequence. We are not able to determine at which point someone knew their FHC versus receiving the CRC screening test, i.e., if FHC occurred first before someone performed the CRC screen or if the test was performed before the person knew their FHC.

## Conclusion

To summarize, a family history of cancer was not significantly associated with having a colon cancer screening test. There is a need for public health advocacy to be directed towards increasing awareness of colon cancer screening among people with a family history of cancer.

## Data Availability

The data is publicly available through the Health Information National Trends Survey (HINTS) 5 cycle 3 via the link https://hints.cancer.gov/data/download-data.aspx after filling data transfer and usable forms.

## Abbreviations

CDC: Center for Disease Control and Prevention
CRC: Colorectal cancer
FAP: Familial adenomatous polyposis
FHC: Family history of cancer
HINTS: Health Information National Trends Survey
HNPCC: Hereditary nonpolyposis colorectal cancer

## References

[1] Rawla P, Sunkara T, Barsouk A. Epidemiology of colorectal cancer: incidence, mortality, survival, and risk factors. Gastroenterology. 2019;14(2):89–103. 10.5114/pg.2018.81072 PMC6791134 31616522

[2] Bibbins-Domingo K, Grossman DC, Curry SJ, Davidson KW, Epling JW, García FAR, et al. Screening for colorectal cancer: US preventive services task force recommendation statement. JAMA. 2016;315(23):2564–75. 27304597 10.1001/jama.2016.5989

[3] Davidson KW, Barry MJ, Mangione CM, Cabana M, Caughey AB, Davis EM, et al. Screening for colorectal cancer: US preventive services task force recommendation statement. JAMA. 2021;325(19):1965–77. 34003218 10.1001/jama.2021.6238

[4] Levin B, Lieberman DA, McFarland B, Andrews KS, Brooks D, Bond J, et al. Screening and surveillance for the early detection of colorectal cancer and adenomatous polyps, 2008: a joint guideline from the American cancer society, the US multi-society task force on colorectal cancer, and the American college of radiology. Gastroenterology. 2008;134(5):1570–95. 18384785 10.1053/j.gastro.2008.02.002

[5] Levin TR, Corley DA, Jensen CD, Schottinger JE, Quinn VP, Zauber AG, et al. Effects of organized colorectal cancer screening on cancer incidence and mortality in a large, community-based population. Gastroenterology. 2018;155(5):1383–91. 30031768 10.1053/j.gastro.2018.07.017 PMC6240353

[6] Wolf AMD, Wender RC, Etzioni RB, Thompson IM, D’Amico AV, Volk RJ, et al. American cancer society guideline for the early detection of prostate cancer: update 2010. CA Cancer J Clin. 2010;60(5):70–98. 20200110 10.3322/caac.20066

[7] Giovannucci E, Rimm EB, Stampfer MJ, Colditz GA, Ascherio A, Kearney J, Willett WC. A prospective study of cigarette smoking and risk of colorectal adenoma and colorectal cancer in US men. J Natl Cancer Inst. 1994;86(3):183–91. 10.1093/jnci/86.3.183 8283490

[8] Henrikson NB, Webber EM, Goddard KA, Scrol A, Piper M, Williams MS, et al. Family history and the natural history of colorectal cancer: systematic review. Genet Med. 2014;17(9):702–12. 10.1038/gim.2014.188 PMC4955831 25590981

[9] Centers for Disease Control and Prevention. Vital signs: colorectal cancer screening test use - United States, 2012. Morb Mortal Wkly Rep. 2013;62(44):881–8. PMC4585592 24196665

[10] Joseph DA, DeGroff AS, Hayes NS, Wong FL, Plescia M. The colorectal cancer control program: partenering to increase population level screening. Gastrointest Endosc. 2011;73(3):429–34. 21353839 10.1016/j.gie.2010.12.027

[11] Rex DK, Johnson DA, Anderson JC, Schoenfeld PS, Burke CA, Inadomi JM. American college of gastroenterology guidelines for colorectal cancer screening 2008. Am J Gastroenterol. 2009;104(3):739–50. 19240699 10.1038/ajg.2009.104

[12] Wilkins T, McMechan D, Talukder A, Herline A. Colorectal cancer screening and surveillance in individuals at increased risk. Am Fam Physician. 2018;97(2):111–6. 29365221

[13] National Cancer Institute. Health Information National Trends Survey 5 (HINTS 5) cycle 3 methodology report. Vol. 5. 2019. p. 82.

[14] National Cancer Institute. Overview of the HINTS 5 cycle 3 survey and data analysis recommendations. 2020. p. 74.

[15] National Cancer Institute. Health Information National Trends Survey 5 (HINTS 5) web pilot results report. Vol. 5. 2021. p. 96.

[16] World Population Review. Divorce rate by state 2022. 2022. Available from: https://worldpopulationreview.com/state-rankings/divorce-rate-by-state.

[17] Ladabaum U, Dominitz JA, Kahi C, Schoen RE. Strategies for colorectal cancer screening. Gastroenterology. 2020;158:418–32. 31394083 10.1053/j.gastro.2019.06.043

[18] White A, Thompson TD, White MC, Sabatino SA, de Moor J, Doria-Rose PV, et al. Cancer screening test use - United States, 2015. Morb Mortal Wkly Rep. 2017;66(8):201–6. 10.15585/mmwr.mm6608a1 PMC5657895 28253225

[19] Sabatino SA, Thompson TD, White MC, Shapio JA, Moor J de M, Doria-Rose VP, et al. Cancer screening test receipt - United States, 2018. Morb Mortal Wkly Rep. 2021;70(2):29–35. 10.15585/mmwr.mm7002a1 PMC7808714 33444294

[20] Hall NJ, Rubin GP, Dobson C, Weller D, Wardle J, Ritchie M, et al. Attitudes and beliefs of non-participants in a population-based screening programme for colorectal cancer. Heal Expect. 2013;18:1645–57. 10.1111/hex.12157 PMC5060871 24268129

[21] Macrae FA, Hill DJ, St. John DJB, Ambikapathy A, Garner JF. Predicting colon cancer screening behavior from health beliefs. Prev Med (Baltim). 1984;13(1):115–26. 10.1016/0091-7435(84)90044-6 6718327

[22] de Moor JS, Cohen RA, Shapiro JA, Nadel MR, Sabatino SA, Robin Yabroff K, et al. Colorectal cancer screening in the United States: trends from 2008 to 2015 and variation by health insurance coverage. Prev Med (Baltim). 2018;112:199–206. 10.1016/j.ypmed.2018.05.001 PMC6202023 29729288

[23] Wee CC, McCarthy EP, Phillips RS. Factors associated with colon cancer screening: the role of patient factors and physician counseling. Prev Med (Baltim). 2005;41(1):23–9. 10.1016/j.ypmed.2004.11.004 15916989

